# Whales, dolphins or fishes? The ethnotaxonomy of cetaceans in São Sebastião, Brazil

**DOI:** 10.1186/1746-4269-3-9

**Published:** 2007-02-20

**Authors:** Shirley P Souza, Alpina Begossi

**Affiliations:** 1 Programa de Pós-Graduação em Ecologia, IB, UNICAMP, C.P.6109, Campinas, SP, 13.083-970, Brazil; 2 Fisheries and Food Institute, Rua Coronel Quirino 1636, Campinas, SP, 13025-002, Brazil; 3 Programa de Capacitação de Pescadores Artesanais para o Manejo da Pesca, PREAC, UNICAMP, Campinas, SP, 13083-970, Brazil; 4 Projeto SOS Mamíferos Marinhos, Instituto Terra & Mar, São Sebastião, SP, Brazil

## Abstract

The local knowledge of human populations about the natural world has been addressed through ethnobiological studies, especially concerning resources uses and their management. Several criteria, such as morphology, ecology, behavior, utility and salience, have been used by local communities to classify plants and animals. Studies regarding fishers' knowledge on cetaceans in the world, especially in Brazil, began in the last decade. Our objective is to investigate the folk classification by fishers concerning cetaceans, and the contribution of fishers' local knowledge to the conservation of that group. In particular, we aim to record fishers' knowledge in relation to cetaceans, with emphasis on folk taxonomy. The studied area is São Sebastião, located in the southeastern coast of Brazil, where 70 fishers from 14 communities were selected according to their fishing experience and interviewed through questionnaires about classification, nomenclature and ecological aspects of local cetaceans' species. Our results indicated that most fishers classified cetaceans as belonging to the life-form 'fish'. Fishers' citations for the nomenclature of the 11 biological species (10 biological genera), resulted in 14 folk species (3 generic names). Fishers' taxonomy was influenced mostly by the phenotypic and cultural salience of the studied cetaceans. Cultural transmission, vertical and horizontal, was intimately linked to fishers' classification process. The most salient species, therefore well recognized and named, were those most often caught by gillnets, in addition to the biggest ones and those most exposed by media, through TV programs, which were watched and mentioned by fishers. Our results showed that fishers' ecological knowledge could be a valuable contribution to cetaceans' conservation, helping to determine areas and periods for their protection, indicating priority topics for research and participating in alternative management related to the gillnet fisheries.

## Introduction

Natural science comprehends the observation and study of the ways in which nature works. Consequently, scientists have gathered an empirical knowledge of the physical and biological world in order to provide a better understanding of the universe. Anthropologists and biologists have been studying 'local' or traditional knowledge accumulated for generations by several communities around the world [1]. The local knowledge about the natural world is the object of study of Ethnobiology, which studies the interactions between human population and natural resources, with special concern to human perception, knowledge and resource uses and management [2]. Human societies depend on natural resources and in this process humans began classifying plants and animals, originating diverse folk taxonomies [3]. The importance of the cognitive process – recognition, categorization and identification – was suggested by several authors [4,5] and Simpson [6] synthesized this point of view in his famous declaration: "*classification... is an absolute and minimal requirement of being or staying alive*". Berlin [3] reinforces this view when he affirms that the human ability to recognize and categorize animals and plants is probably innate as we have an unconscious perception of the biological reality. Mishler and Donoghue [7] also argument that human brains are linked to the same neural process of "grouping by perception".

The reality of biological species has been discussed since Lamarck and Lyell in the 18^th ^century [8] and Darwin [9] in "The Origin of Species" questions about the reality of species when he points out that the term species is arbitrary. Nevertheless, in unpublished notes from 1871, Darwin accepted the idea of biological discontinuities. Such discrete groups among plants and animals were considered by Dobzhansky as universal, a fundamental characteristic of biological diversity [10].

Several criteria, such as morphology, ecology, behavior, utility and salience ('biological distinctiveness'), have been used by local communities to classify plants and animals [3,11,12]. The process of classifying and giving names to plants and animals was extensively studied by Berlin [3] who defines general principles to ethnobiological categorization and nomenclature. Hunn [13] suggests that cultural knowledge must be useful, or adaptive, considering the amount of energy invested in obtaining it. According to this author '*human perception is programmed to recognize patterns among living organisms*' [13].

Local ecological knowledge (LEK), also known as traditional ecological knowledge (TEK), has been studied in several parts of the world, not only with the purpose of retrieving or bringing value to vanishing cultures but as a useful tool to improve natural resources' conservation and management policies [14-17]. Furthermore, LEK involves not only ecological knowledge accumulated and community's beliefs, but also its social systems of rules necessary to manage local resources, which are transmitted through generations by culture [18].

Besides other traditional or 'local' communities, fishers' groups have been studied in several countries around the world, especially in relation to their knowledge about plant's utilization, fish ecology and fisheries management [11,14,15,17,19-30]. Studies regarding fishers' knowledge about cetaceans in the world, with special reference to Brazil, began in the last decade [31-36] and are mostly related to the ecological aspects. The frequent occurrence of whales and dolphins in the northern coast of São Paulo State (Ubatuba, Caraguatatuba, São Sebastião and Ilhabela municipalities) has been confirmed along the last 12 years, through records of sightings and stranded or incidentally captured animals. According to reports of the '*Projeto SOS Mamíferos Marinhos*', from September 1994 to September 2006, 138 cetaceans of 16 species (*Megaptera novaeangliae*, *Balaenoptera edeni*, *B. acutorostrata*, *Eubalaena australis*, *Pontoporia blainvillei*, *Sotalia guianensis*, *Stenella frontalis*, *Tursiops truncatus*, *Steno bredanensis*, *Delphinus capensis*, *D. delphis*, *Kogia sima*, *Pseudorca crassidens*, *Orcinus orca*, *Berardius arnouxii and Mesoplodon mirus*) have been recorded dead or alive, in the studied area (S.S. unpublished data 2006).

Artisanal fisheries are one of the main commercial activities practiced by local communities, called '*caiçara*', living in coastal sites of the Atlantic Forest, in Brazil. In previous surveys carried out by '*Projeto SOS Mamíferos Marinhos*' one of the authors (S.S.) recorded the interactions between fishers and cetaceans, especially in relation to species of coastal dolphins which occur at the main fishing points used by fishers and that are occasionally caught by gillnets. Two of these species, *Pontoporia blainvillei *and *Sotalia guianensis *are the most impacted by incidental catch and the former one is considered vulnerable, according to IUCN and IBAMA red lists [37,38]. The International Whaling Commission (IWC) has recognized, in 1972, the accidental capture of cetaceans as a threat to populational stocks of small cetaceans, especially from the families Phocoenidae, Pontoporiidae and Delphinidae [39].

Our objective, in this study, is to record the fishers' knowledge in relation to cetaceans, with special emphasis on folk taxonomy (ethnotaxonomy), analyzing fishers' forms of classification and nomenclature of whales and dolphins in the Southeastern Brazilian coast. We expect to find a detailed nomenclature among fishers, especially related to dolphins' species, since these animals are frequently observed by fishers at sea and some of them are incidentally captured along coastal beaches.

## Materials and methods

### Studied Area

**Figure 1:**
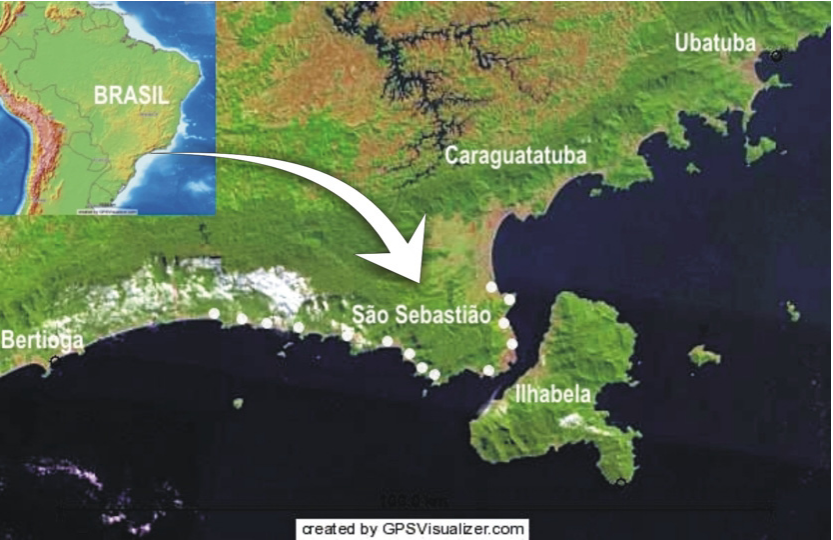
Northern coast of São Paulo State, showing fishers' communities studied (white dots) at São Sebastião.

The northern coast of São Paulo State is 161 km long and it is composed by 164 beaches and 17 islands. It encompasses the districts of Ubatuba, Caraguatatuba, São Sebastião and Ilhabela (Figure 1). Currently, tourism is the most important commercial activity in the region as a whole. The district of São Sebastião (23°42'18" to 23°45'38"S – 45°25'41" to 45°53'49"W) is composed by a narrow plain area located between the sea (Atlantic Ocean) and the slopes of Atlantic Forest and is inhabited by nearly 70.000 people [40]. Its coast line is 80 km long and is composed by 34 beaches. The biggest oil terminal (TEBAR – TRANSPETRO) of Latin America is located in the town and is the main source of income to the city of São Sebastião, followed by tourism and fisheries.

### Fisheries activities

**Figure 2:**
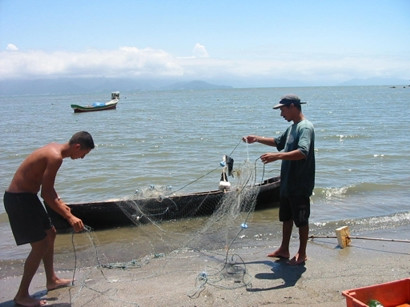
Fishers of Enseada beach, taking a gillnet out of their canoe (São Sebastião, Brasil).

Most of the fishers in São Sebastião, as well as from other sites of the northern coast of the São Paulo State, practice artisanal coastal fisheries, using paddled canoes or motored boats measuring from 5 to 15 meters. Trawling nets, several kinds of gillnets and hook and line are the main equipment used in the local fisheries (Figure 2). Artisanal fishing is practiced by nearly 250 members of communities around the coast of S. Sebastião, but according to elders, the involvement of local people in such activity has been decreasing over the years. One of the reasons for that could be the reduced monetary incomes earned from fishing activities year by year as a result of a probable fish stock depletion, among other causes.

### Methodology

Fishers resident in 14 communities along São Sebastião coast were selected according to criteria related to local fishing experience: age more than 35 years, living in the studied area for more than 10 years and fishing, as main activity, for more than 15 years. A total of 70 fishers were selected. Some of them had participated in previous interviews about the interaction cetacean versus fisheries and were known by their confirmed experience in fisheries. Others were indicated by these ones through the 'snow-ball' method, used in other studies [41,42]. When asked about his accordance in participating of the interviews, each selected fisher gave his consent. The communities chosen were: Enseada, Cigarras, São Francisco, Pontal da Cruz, Barequeçaba, Toque-Toque Grande, Toque-Toque Pequeno, Paúba, Maresias, Boiçucanga, Barra do Sahy, Juqueí, Barra do Una and Boracéia (Figure 1). The interviews were carried out from January 2005 to July 2006.

Using partially structured questionnaires, as well as unlabelled figures and photos of 11 cetaceans' species which occur in the studied area, we interviewed each fisher individually, asking questions about cetaceans' names, classification and the form by which they could be grouped. Cetaceans' species included in this survey were: Family Balaenidae: *Eubalaena australis*, Family Balaenopteridae: *Megaptera novaeangliae*, *Balaenoptera edeni *and *B. acutorostrata *from the suborder Mysticeti and from the suborder Odontoceti, Family Delphinidae: *Orcinus orca*, *Tursiops truncatus*, *Steno bredanensis*, *Stenella frontalis*, *Delphinus *sp., *Sotalia guianensis *and Family Pontoporiidae: *Pontoporia blainvillei*. The distribution of these species along São Sebastião coast is heterogeneous, some of them occurring at the northern coast, others occurring at the southern coast and some have been recorded at the entire coast [43-46].

In previous researches on cetaceans at the studied area along the last 12 years, we had perceived that a significant number of local fishers referred to whales and dolphins as 'fishes'. In face of this trend, we decided to ask two direct questions related to cetaceans' classification ('*Are whales and dolphins fish*?' and '*If yes*/*no*, *why?*'), in order to quantify the proportion of fishers who considered (or not) cetaceans as fishes and to find out what other classification groups that they could eventually mention.

In order to obtain the names used by the local fishers regarding to the studied cetaceans, we asked the questions '*Do you know this animal?*' and '*How do you call it?*'

All fishers were interviewed when working or staying at their '*ranchos*', places where they keep their fishing equipments and where they can be found before or after going to sea. Interviews had an average duration of 45 minutes. After fishers' consent, we took photos of their equipments, boats and activities during the interviews, in order to illustrate the local fisheries.

### Data analysis

The answers given by the fishers were recorded as 'citations', being possible to obtain more than one citation per answer relative to each question. Therefore, for some questions the number of citations was different from the number of interviewed fishers. In order to standardize the results, we show the data as the total number of citations for each question, followed by its correspondent percentage value when necessary. We included all the citations, not only those most mentioned by fishers, to avoid loosing rare or uncommon names but statistical analyses did not consider citations with very low frequencies (5 or below).

Cetaceans' species identification and its English common names followed [47]. We adopted Berlin's ethnobiological classification, which recognizes 6 hierarquical groupings (*taxa*) of greater and lesser inclusiveness called *kingdom*, *life-form*, *intermediate*, *generic*, *specific *and *varietal *[3]. In this study we compared scientific ranks (suborders Mysticeti and Odontoceti and species) to folk ranks (generic and specific) and identified possible correspondences among Linnaean and folk categories. The types of correspondence between scientific and folk taxonomies followed Berlin [48].

Concerning classification of cetaceans we tested citations obtained for the different categories using Chi-square to see if there is any difference between whales and dolphins' classifications. However, we analyzed citations related to killer whales (*Orcinus orca*) into the group 'whales', because despite being an Odontoceti from the Family Delphinidae, represented mostly by dolphins and porpoises, *O. orca *is considered a whale by many cultures around the world, due to its bigger size in relation to other dolphins. Nevertheless in relation to nomenclature we analyzed killer whales apart, due to the external factors that influence its nomenclature, not including this species in the statistical analyses performed.

We tested nomenclature's citations for two geographic areas – northern and southern coast of São Sebastião – according to the localization of the studied fishers' communities, through Chi-square test, in order to verify possible patterns of distribution among cetaceans' species along the coast.

The frequency of citations for the 6 most quoted species and the frequency of records (related to incidentally caught or stranded individuals in the studied area, data gathered from a 12 years' research by 'Projeto SOS Mamíferos Marinhos') were compared using Chi-square test, in order to see if species are cited in the same proportion in which they are captured or appear dead on the beaches. All statistical tests were performed using the software BioEstat 4.

## Results

Among the interviewees, 46 (66%) are native from São Sebastião, 10 (14%) are from neighboring municipalities such as Caraguatatuba, Ilhabela and Santos, and 14 (20%) are from inland cities of São Paulo or from other states. There was just one woman among the 70 fishers interviewed. Ages varied from 35 to 97 years, average age being 59 years old. The average period dedicated to fisheries, among all the interviewees, was about 40 years. The minimum time of residence in São Sebastião was 13 years and the maximum was 97 years. Regarding to time attending a school, 4 (6%) never went to school, 37 (53%) attended primary school but only 19 finished it, 31 (44%) started secondary school but only 4 finished it, 7 (10%) completed high-school, 1 started college but did not finish it and 9 (13%) did not know how to answer this question.

### Classification

Regarding to the questions about cetaceans' classification, some fishers did split their answers related to whales or dolphins, others answered by grouping whales and dolphins together. We separated the answers relative to whales and dolphins in order to better understand the variations in the number of citations relative to each mentioned category.

**Figure 3:**
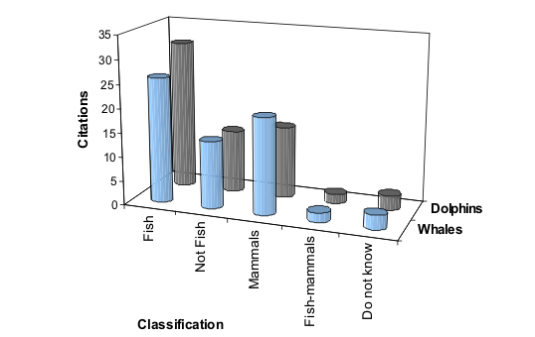
Whales' and dolphins' classification according to fishers (n = 70) from São Sebastião, Brazil.

In relation to whales, in 26 citations (37%) fishers considered them as fish. The other citations consider whales as mammals (28%) and as 'non-fish' (20%), but did not define them as mammals (Figure 3). Regarding dolphins' classification, according to fishers' answers, almost half of the citations (31 or 44%) mentioned that dolphins are fish, while 21% considered them as mammals and another 18% affirmed that they are not fish (Figure 3). Statistically, there was no difference among the answers referent to the three life-form categories ('fish', 'mammal', 'not-fish') for whales or dolphins (χ^2 ^= 1.18, d.f. = 2, p = 0.55) (Table 1).

**Table 1:** Contingency table using the number of citations for life-form categories quoted for whales and dolphins.

Life-form Categories	Whales	Dolphins	χ^2^	*P*
Fish	26	31		
Not Fish	14	13		
Mammal	20	15	1.182	0.554*

Notes: Ho: proportion of citations for 'Fish', 'Not Fish' and 'Mammal' categories are independent if the animal is whale or dolphin.

* Non-significant *P *value, Ho is not rejected.

It was interesting to note that for the three categories ('fish', 'mammal', 'not fish') mentioned above, we found the subcategory "*look like sharks*/*are from the sharks' family*" as an additional answer, represented by 8 citations for whales as well as for dolphins. Two fishers considered whales and dolphins as a mixed category called 'fish-mammals' and three fishers did not know how to classify these animals.

### Nomenclature

**Figure 4:**
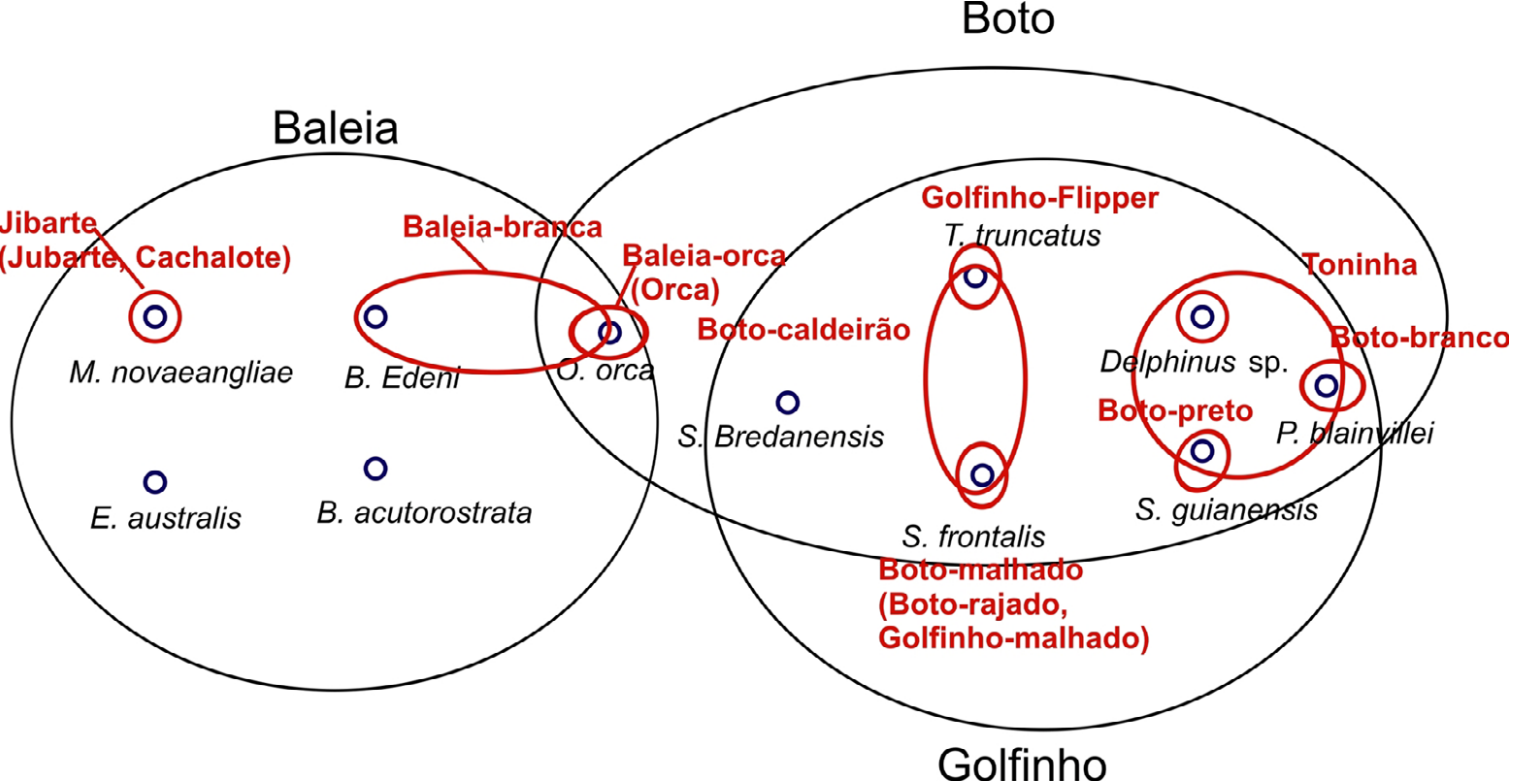
Cetaceans' classification, according to fishers from São Sebastião, Brazil. Blue circles correspond to biological species, red circles/ellipses are folk species and black ellipses correspond to generic rank.

**Figure 5:**
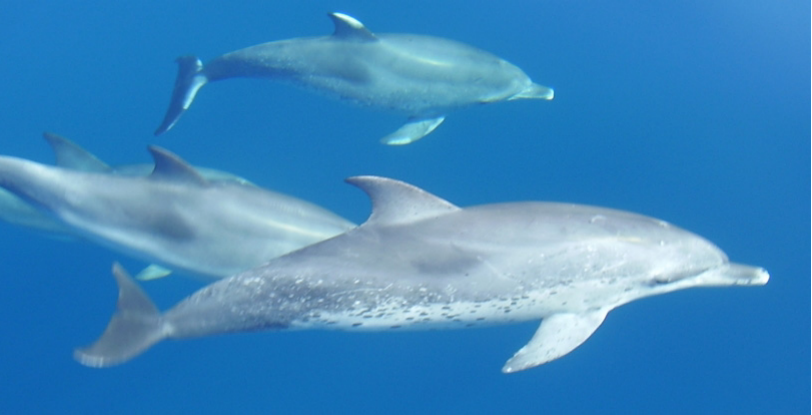
Atlantic spotted dolphins swimming near to the Vitória Island, Ilhabela, Brazil. Note the spotted pattern on the ventral side of the animal. (Photos by 'Projeto SOS Mamíferos Marinhos')

From fishers' answers related to nomenclature we obtained 3 generic names and 14 folk species, 9 of them corresponding to binomials (Figure 4). Most of these binomials were composed by a describer related to the animal's coloration pattern (Table 2). We also verified two types of correspondence between scientific and folk systems of classification (Table 2). The first was 'Over-differentiation type I', when two or more folk taxa refer to a single biological species, as in the case of the folk species 'jibarte' and 'jubarte' referring to *Megaptera novaeangliae*, 'orca' and 'baleia orca' referring to *Orcinus orca *and 'boto-rajado', 'boto-malhado' and 'golfinho-malhado' referring to *Stenella frontalis *and its spotted coloration pattern (Figure 5). The second type of correspondence was 'Under-differentiation type II', when a single folk genera refers to two or more species of two or more genera, as in the case of 'baleia', quoted for 3 species of whale (order Mysticeti) and *for O. orca *(order Odontoceti), 'baleia-branca' mentioned to *B. edeni *and for *O. orca*, 'boto' quoted for 7 species of dolphins (order Odontoceti), 'golfinho' mentioned for 6 species of dolphins, and folk species 'toninha' cited for 3 species of dolphins and 'boto-caldeirão' quoted for 2 species of dolphin.

**Table 2:** Cetaceans' names given by the fishers interviewed (n = 70).

**Scientific name**	**English common name**	**Folk genera**	**Folk species**	**Number of citations**	**Fisher do not know the animal**	**Number of fishers who have seen the animal (S) or have not seen the animal (NS)**	
*Eubalaena australis*	Southern right whale	**baleia**		**37**	33	S = 37	NS = 33
*Balaenoptera acutorostrata*	Minke whale	**baleia**		**1**	69	S = 1	NS = 69
*Balaenoptera edeni*	Bryde's whale	**baleia**		**19**	52	S = 18	NS = 52
			baleia-branca	1			
*Megaptera novaeangliae*	Humpback whale	**baleia**		**13**	33	S = 37	NS = 33
			jibarte	15			
			jubarte	8			
			cachalote	2			
*Orcinus orca*	Killer whale	**baleia**		**27**	25	S = 14	NS = 56
			orca	15			
			baleia-orca	12			
			baleia-branca	1			
		**boto**		**2**			
*Steno bredanensis*	Rough-toothed dolphin	**boto**		**14**	54	S = 16	NS = 54
		**golfinho**		**2**			
*Tursiops truncatus*	Bottlenose dolphin	**boto**		**56**	7	S = 63	NS = 7
			boto-caldeirão	9			
		**golfinho**		**10**			
			golfinho-flipper	1			
*Delphinus *sp.	Common dolphin	**boto**		**17**	50	S = 20	NS = 50
		**golfinho**		**1**			
			toninha	2			
*Stenella frontalis*	Atlantic spotted dolphin	**boto**		**25**	43	S = 27	NS = 43
			boto-rajado	2			
			boto-caldeirão	1			
			boto-malhado	1			
		**golfinho**		**2**			
			golfinho-malhado	1			
*Sotalia guianensis*	Marine Tucuxi	**boto**		**45**	8	S = 61	NS = 9
			boto-preto	1			
		**golfinho**		**12**			
			toninha	8			
*Pontoporia blainvillei*	Franciscana		toninha	51	6	S = 64	NS = 6
		**boto**		**6**			
			boto-branco	1			
		**golfinho**		**14**			

Concerning to whales, including *O. orca*, the folk name most cited was 'baleia', with 85 citations (98% of the generic names mentioned for whales), followed by folk species 'orca' (27 citations) and 'jibarte' (15 citations) (Table 2).

In relation to *O. orca*, many fishers who knew its name said to have learnt it from TV programs. Although it's not a common species in the studied area, 45 fishers (64%) cited 2 generic names ('baleia' and 'boto') and 3 binomials ('orca', 'baleia-orca' and 'baleia-branca') referring to killer whales and among them only 12 have seen this animal at sea (Table 2). The other 33 interviewees have only seen it on TV.

In the case of dolphins' species, the generic name most quoted was 'boto', with 165 citations (59% of the generic names quoted for dolphins), followed by folk species 'toninha' (61 citations) and generic name 'golfinho' (41 citations) (Table 2).

From the 10 cetaceans species occurring at the coast of São Sebastião, excluding *Orcinus orca*, which was analyzed apart in relation to nomenclature, 9 species were cited by fishers from the northern coast and 10 by those from the southern coast. Nevertheless, when compared through Chi-square test, data showed no differences between the frequency citations for each species between these two areas (χ^2 ^= 4.17, d.f. = 8, p = 0.84) (Table 3).

**Table 3:** Contingency table using the number of citations for each cetacean species quoted for the Northern and Southern coast of São Sebastião.

Cetacean Species	Northern coast	Southern coast	χ^2^	*P*
*E. autralis*	13	24		
*B. edeni*	9	9		
*M. novaeangliae*	16	21		
*T. truncatus*	31	32		
*S. bredanensis*	8	8		
*Delphinus *sp.	11	9		
*S. frontalis*	13	14		
*S. guianensis*	32	30		
*P. blainvillei*	27	37	4.172	0.8413*

Notes: Ho: proportion of citations for each species is independent if the fisher is from the Northern or the Southern coast of São Sebastião. * Non-significant *P *value, Ho is not rejected.

The frequency of citations for *M. novaeangliae*, *T. truncatus*, *S. bredanensis*, *S. frontalis*, *S. guianensis *and *P. blainvillei *were compared to the frequency of records for those species through Chi-square Tests, which showed that numbers of species' citations are very different to those of species' records in the studied area indicating that there is no proportionality between citations and occurrences of incidental capture or stranding of cetaceans (Table 4).

**Table 4:** Citations of the most frequent cetaceans' species according to fishers from São Sebastião and records of the same species incidentally captured or stranded in the studied area (from September 1994 to September 2006).

**Cetacean species**	Records of dead cetaceans (1994–2006)*	**Cetaceans' citations by fishers**	χ^2^	*P*
*M. novaeangliae*	6	38	23.273	< 0.0001**
*T. truncatus*	14	66	33.80	< 0.0001**
*S. bredanensis*	6	16	4.545	0.0550
*S. frontalis*	8	27	10.314	0.0023
*S. guianensis*	30	65	12.895	0.0005**
*P. blainvillei*	49	71	4.033	0.0552
Total	113	283		

*Data from 12 years of monitoring in the studied area by 'Projeto SOS Mamíferos Marinhos' team (S.S. unpublished data, 2006)

Notes: Ho: number of citations is proportional to the number of records for each species. **Significant *P *value, Ho is rejected.

### Clustering the studied species by similarity

**Figure 6:**
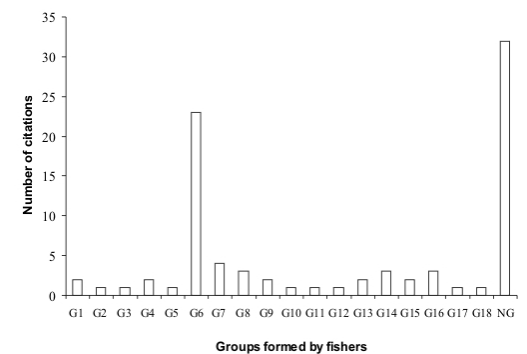
Groups of cetaceans' species formed by fishers, according to morphological similarities. (G1 = *E. australis *+ *M. novaeangliae*, G2 = *E. australis *+ *M. novaeangliae *+ *O. orca*, G3 = *B. acutorostrata *+ *M. novaeangliae*, G4 = *B. edeni *+ *B. acutorostrata*, G5 = *B. edeni *+ *B. acutorostrata *+ *O. orca *+ *S. bredanensis*, G6 = *S. guianensis *+ *T. truncatus*, G7 = *S. guianensis *+ *T. truncatus *+ *Delphinus sp*., G8 = *S. guianensis *+ *T. truncatus *+ *P. blainvillei*, G9 = *S. guianensis *+ *T. truncatus *+ *S. frontalis*, G10 = *S. guianensis *+ *Delphinus sp*., G11 = *P. blainvillei *+ *Delphinus sp*., G12 = *S. bredanensis *+ *Delphinus sp*., G13 = *S. frontalis *+ *Delphinus sp*., G14 = *S. bredanensis *+ *S. frontalis*, G15 = *S. bredanensis *+ *T. truncatus*, G16 = *S. guianensis *+ *P. blainvillei*, G17 = *P. blainvillei *+ *S. bredanensis*, G18 = *P. blainvillei *+ *T. truncatus*, NG = do not group)

Eighteen clusters (or groups) of the studied species were formed by 63% of the interviewed fishers, based mainly in morphological similarity among species. The most cited one was the group composed by *Tursiops truncatus *and *Sotalia guianensis*, which was cited by 23 fishers (27%). These two species were also present in groups formed by 3 species, mentioned in other 9 citations. Another 32 fishers (37%) answered that it was not possible to group any species, because each one was unique and different from the others (Figure 6).

## Discussion

Folk taxonomy is a form of organizing local communities' knowledge and it may represent different behavioral responses of people related to the salience of each organism [49]. Concerning the interviewed fishers, despite their level of formal education, their long-life accumulated knowledge on marine environment seems to be sophisticated, at least considering local fish species, to which they show knowledge comparable to other studied communities in the southeastern Brazilian coast [10,16,21,22]. On the other hand, cetaceans' folk taxonomy built by fishers living at São Sebastião seems to be a small inventory. The ethnobiological classification of local communities in general is usually based on the salience of the organisms in the local habitat and on the observed morphological and behavioral similarities and differences among the recognized groups [3]. The criteria utilized in cetaceans' classification by the studied fishers are probably related to cognitive aspects. As whales and dolphins are not targets to the fisheries practiced in studied areas, utility may not be the main criterion adopted by fishers in classifying these animals. However whales and dolphins' presence is very salient to be unnoticed, due to their size and behavior.

According to Dougherty [50] the salience of a biological group of organisms results from the degree of direct interaction between people and these organisms. Thus, ethnotaxonomy also reflects the availability of living organisms in the environment [10]. Salience can be cultural as well as perceptual or phenotypic [12] and generally the most salient organisms are named through primary lexemes [51]. This author pointed out that people's vocabulary is related to their long-term interests and the subsistence mode is the main factor determining the size of a folk taxonomy. When societies shift from a non-cultivator (as hunting-gathering ones) to cultivator mode of subsistence, their taxonomies tend to increase as they will be in contact with a new variety of introduced domesticated species, which will be added to the local organisms normally collected or hunted, what promotes the increasing of binomially named species [51,52]. Regarding to their subsistence mode, fishers' communities from São Sebastião could be classified as small-scale cultivation societies, whose taxonomies are characterized by bigger inventories with more binomials if compared to the taxonomies of hunting/gathering societies, which have smaller inventories of highly salient organisms.

The results of interviews showed that whales and dolphins were classified by most fishers into the category 'fish', but with little difference for the other categories ('Mammals' and 'Not fish') (Figure 3). The category 'fish' represents a life-form, which corresponds to a high-ranking folk taxonomic category, following Berlin [3]. Previous researches in ethnotaxonomy have suggested that life-forms may be not always 'natural' groupings [3], being sometimes arbitrary. In spite of being highly salient animals, mammals are not classified into a single life-form in several folk taxonomies [3,53]. Cetaceans, classified as mammals by Linnaean taxonomy, are in fact members of life-form 'fish' in several folk taxonomies, such as those of fishers from Hong Kong, Solomon Islands, Brazilian Northeastern and Southeastern coasts [19,23,24,32]. The life-form 'fish' is characterized by different groups of animals that live in aquatic habitats, including fishes, aquatic invertebrates, turtles, crocodiles, dugongs, whales and dolphins [13,18,19,32]. Paz and Begossi [54] studying the folk taxonomy of fishes at Gamboa, (Sepetiba Bay, Southeastern Brazilian coast) also found that local dolphins are classified as an ethnospecies ('boto') belonging to the ethnofamily 'Cação' (sharks).

Analyzing the life-form 'fish', Hunn [13] considered that it is defined not in terms of morphological similarity as other life-forms, but rather in terms of habitat. Our results agree with his analysis as the main reason mentioned by fishers to classify cetaceans as fish was '*because they live in the sea*' (n = 15). Nevertheless, fishers have the perception that whales and dolphins are '*a different kind of fish*', and the differences most cited were their behavior and the quality of their flesh. In two citations, fishers suggested that these animals are in fact '*fish-mammals*', an idea suggested before by Dupré [55], who discussed the scientific pluralism in biological classification. This author suggests that there are many forms of classification, most of them being arbitrary and overlapping. Life-form 'mammal', the second most quoted by fishers (n = 35) was justified by them in terms of behavioral aspects of cetaceans: '*they are mammals because the mothers breast-feed their calves*', emphasizing the maternal care. This life-form is also considered 'artificial' or arbitrary by Atran [53], because the wide variation of features presented by their representatives' groups makes it difficult for a non-biologist to form a perceptual reference of the life-form 'mammal'.

The concept of cetaceans belonging to the life-form 'fish' is more than a question of perception; it is also part of the process of cultural transmission through generations of studied fishers. This became evident when some fishers mentioned that despite having watched on TV programs the information that cetaceans are in fact mammals, they continue referring to whales and dolphins as fishes because they have learned it from the elders. Cultural transmission from modern societies to local communities was detected too. According to 47% of the interviewed fishers they have learnt about killer whales (*Orcinus orca*) regarding to nomenclature, appearance and behavior watching TV programs, despite have never seen this cetacean in nature before. Independently of the cetacean being classified as 'fish', 'mammal' or just 'not fish', the link between cetaceans (especially dolphins) and sharks was mentioned by eight fishers. The main reason for this association, according to the interviewees, is the morphological similarity between the two groups, especially in relation to the dorsal fin and flippers, texture and color of skin. Their resemblance can be explained by convergent evolution, where different species subjected to similar selective pressures develop similar traits and in their case resulted in adaptation to live in aquatic environments [56]. Both groups occupy the top of the marine trophic web, which contributes to their high natural salience. The perception of the similarity between sharks and cetaceans by the fishers reinforces the inclusion of whales and dolphins in the life-form 'fish'.

When comparing citations with records (stranding and incidental capture) of the six most frequent species (Table 4), we expected that species which occur more frequently would be more recognized and named [57]. In general, this trend was confirmed, but values found for each species' citations were not proportional to the numbers of each species records. This can be explained since not all the species show the same salience, especially if we consider that the probability to be captured in gillnets is greater for the smaller dolphin species [58,59]. Additionally, it is virtually impossible to record all the individuals that die along the year in the area, because only part of the carcasses arrives to inhabited points of the coast. Among dolphins, two of the most cited species (*P. blainvillei *and *S. guianensis*) are the most locally caught by gillnets [44], which increases their salience to the fishers. Concerning whales, in spite of showing seasonal occurrence, lower records of stranding and not being so frequently affected by gillnets as dolphins, they are almost as salient as dolphins, considering fishers' citations.

Our results indicated that fishers from São Sebastião perceive cetaceans primarily as composed of three wider groups, the folk genera 'baleias' (whales), 'botos' and 'golfinhos' (the last two genera referring to dolphins), which are intimately linked by an overall morphological similarity. These folk names were the most readily recognized by fishers, when considering the taxon Cetacea, and included several folk species, some of them binomially named (Figure 4), which agrees with the ethnobiological categorizarion proposed by Berlin [3]. However, this author observed that nearly 80% of folk genera in typical folk taxonomies are monotypic, including no subgeneric taxa [3]. This observation did not apply to our data, since the 3 folk genera are not monotypic.

Whales were named by the interviewed fishers mostly by the folk genera 'baleia', which could reflect its low salience as species. A possible explanation could be its rarer presence along the year, showing peaks of occurrence during the winter [45], when fisheries is more affected by the bad weather, decreasing the oportunity of encounters between whales and fishers. Specific names were mentioned by fishers especially for the two species most exposed by TV programs (humpback and killer whales), denoting external influences on the fishers' perception in the studied region. Humpback and killer whales are between the most studied cetaceans' species in the world [37,38] and TV documentaries about them are frequently showed in the most watched Brazilian TV Channel (Globo TV). Killer whale (*O. orca*), despite being an Odontoceti, was included by fishers in the generic tank 'baleia' (which includes all the Mysticeti). It is a large animal, comparable to the smaller species of whales. Hunn [57] pointed out that size is an important characteristic directly related to perceptual salience, increasing the chances of an organism to be recognized. In fact, for fishers from São Sebastião, *O*. orca figured at the boundary between whales (Mysticeti) and dolphins (Odontoceti), which could be explained by the fact that fishers perceive not only killer whale's size as well as its behavior, which remembers dolphins' behavior.

The specific name 'jibarte' quoted for *Megaptera novaeangliae *was slightly more used than the generic name 'baleia', confirming this species of Mysticeti as the most recognized by local fishers. The synonim 'jubarte' was probably acquired from external influences (TV programs or researchers' talk) and the specific name 'cachalote', which is the common name for *Physeter macrocephalus *(not included in this survey) was quoted only for the males of *M. novaeangliae*, an information learned from the 'elders', according to the interviewed fishers (n = 4).

'Boto' and 'golfinho' were quoted by interviewed fishers as generic names for dolphins in general. Dolphins' common names show high geographic variation, even those used in scientific texts [34,35,60]. Many times one common name is used for different species, in different regions of Brazilian coast, as in the case of 'boto' and 'golfinho', which can be common names for *Tursiops truncatus*, *Sotalia guianensis*, *Stenella frontalis *and other species of delphinids. The use of 'boto' was four times more common among the interviewed fishers than the use of 'golfinho'. Generally 'golfinho' is preferred by scientific jargon and communication media. When using both names, few interviewed fishers (n = 5) associated 'boto' to more robust dolphins and 'golfinho' to slimmer ones. Fishers from Cananéia mentioned the same association between generic names and dolphins' size [35].

**Figure 7:**
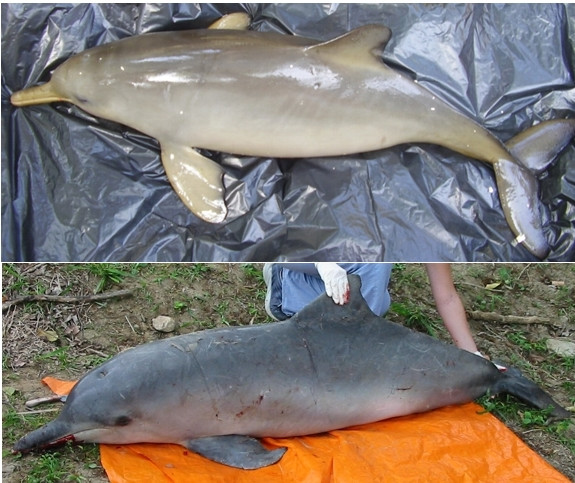
A female of *Pontoporia blainvillei *(top) and a male of *Sotalia guianensis *(bottom) incidentally caught by gillnets at São Sebastião, Brazil. (Photos by 'Projeto SOS Mamíferos Marinhos')

The mention of the specific name 'toninha' for three different dolphins' species can be explained by morphological and behavioral similarity in the case of *S. guianensis *and *P. blainvillei*, as both species are the smaller ones and occur in coastal habitats in the studied area (Figure 7). *Delphinus *sp., despite its bigger size, can be considered a slimmer dolphin, which could lead some fishers to perceive it as a 'small' dolphin. On the other hand, the association of 'toninha' to *Delphinus *sp. could result from a mistaken identification of this dolphin's picture, since this species is relatively rare in the surveyed area, so it could have been confused with other common species. The use of the name 'toninha' shows great variation along the Brazilian coast. In the south of Brazil it generally refers to *Pontoporia blainvillei *[32,35,44]. However for the State of Espírito Santo (Brazilian southeastern coast) Freitas-Netto [60] reports its use for dolphin species of greater size such as *Steno bredanensis*, *Tursiops truncatus *and those of the genus *Stenella*.

Begossi *et al. *[10] studying the ethnotaxonomy of fishes on the Brazilian Atlantic Forest coast, compared generic richness of fishes with citations '*I do not know*' and found that the diversity of folk genera cited increases as the fishers show less knowledge about the fishes. Concerning cetaceans' taxonomy, citations '*I do not know*' showed great variation among the studied species, varying from 8% for *P. blainvillei *(the most known species) to 98% for *B. acutorostrata *(the least known species), indicating heterogeneity in relation to fishers' knowledge about these species.

A group among the interviewed fishers formed one main cluster, including two biological species (*T. truncatus *and *Sotalia guianensis*), which were grouped by morphological similarity (23 citations, 27% of the total). These species are in fact morphologically very similar to any non-biologist, differing only in relation to their size and minor morphological aspects, such as the coloration pattern and the shape of their head, flippers and dorsal fins. Jefferson *et al. *[47] mentioned that individuals of *T. truncatus *can be confused along the east coast of South America with dolphins of the genus *Sotalia*. On the other hand, another group of the interviewees (37%) did not find enough similarity among the studied species in order to group them. This showed a great variation relative to the perception between these two groups of fishers which deserves further investigation, to clarify why fishers living in the same area and exposed to the same cetaceans' species have different perceptions in relation to morphological similarity among these species.

## Conclusion

Cetaceans' genera normally include few Linnnean species, but their families include genera showing a high morphological similarity, which makes species recognition in nature a difficult task, succesfully performed only by cetaceans' experts. Fishers' perception about cetaceans was highly influenced by phenotypic and cultural salience of the whales and dolphins, since fishers did not see these animals from a utility point of view. Historically, the utilization of accidentally captured cetaceans has not occurred in the studied area, according to interviewed fishers'. Thus, phenotypic salience favored cetaceans' recognition as natural discontinuities in nature, supporting, in some cases, the reality of the species ('toninha', 'jibarte', 'orca', 'boto-caldeirão').

Our results indicated that the most salient were not necessarily the most abundant species, but included the most frequently caught ones (*P. blainvillei *and *S. guianensis*) and those of greater size (although being rare), such as *T. tursiops *and *E. australis*. These species, together with those exposed by media (*M. novaeangliae *and *O. orca*), were the most recognized and important ones in the process of classification and nomenclature by the interviewed fishers, being named to the folk species level.

Coincidently, some of these species are the most threatened in the surveyed area, but not enough studied, which makes the fishers' knowledge about them greatly valuable for their conservation. According to Berkes *et al. *[18] local ecological knowledge can provide management alternatives to cope with dynamic changes in ecosystems, contributing to the conservation of marine habitats. Our results showed that fishers' LEK could be used to indicate areas and seasons of great occurrence of vulnerable cetaceans' species, helping to determine areas and periods for their protection. As researches about cetaceans' biology are of long-term duration and generally expensive, fishers' knowledge could also indicate priority topics for research, especially in those regions of the coast where no research has been conducted. On the other hand, fishers' LEK seems to be increasingly influenced by media, risking becoming global and loosing important social mechanisms of cultural transmission. However, some change is expected in the studied fishers' knowledge due to the increasing contact among them and the emergent tourism, what not necessarily means a negative experience. As fishers demonstrated empathy for cetaceans, maybe the contact with updated information could increase fishers' awareness about the threatens to cetaceans', promoting their cooperation in the conservation of these species through an alternative management of gillnet fisheries in order to minimize incidental captures.
